# The Worth of Words

**Published:** 1903-12

**Authors:** 


THE WORTH OF WORDS. By Dr. Raley Husted Bell. With an introduction by Dr. William Colby Cooper. Third edition; revised and enlarged. Published by Hinds & Noble, 31-35 W. Fifteenth street, New York city.

No class of men in our elaborate social system is more in need of a select and elegant vocabulary than the modern practicing physician. His position in the social world is unique. Living as he does midway between the abstract truths of science on one hand and the popular conception of things on the other, he must be at once a philosopher and an interpreter. How often is he called upon to explain the nature of a patient's malady, to discuss some recent invention or discovery, to give advice, to warn. He must be equally at home at a banquet, with the sick in the slums or at the bedside of an elegant and sensitive woman. He must talk always—and he must please always. In conversation he must know how to be chaste, elegant, precise, cautious,—in short, he must know "The Worth of Words." J. M. L.

